# High-Sensitivity Encoder-Like Micro Area-Changed Capacitive Transducer for a Nano-g Micro Accelerometer

**DOI:** 10.3390/s17092158

**Published:** 2017-09-20

**Authors:** Wenjie Wu, Panpan Zheng, Jinquan Liu, Zhu Li, Ji Fan, Huafeng Liu, Liangcheng Tu

**Affiliations:** 1 MOE Key Laboratory of Fundamental Physical Quantities Measurement & Hubei Key Laboratory of Gravitation and Quantum Physics, School of Physics, Huazhong University of Science and Technology, Wuhan 430074, China; wjwu@hust.edu.cn (W.W.); D201177044@hust.edu.cn (P.Z.); jinquanliu@hust.edu.cn (J.L.); lizhu@hust.edu.cn (Z.L.); fanji@hust.edu.cn (J.F.); huafengliu@hust.edu.cn (H.L.); 2 Institute of Geophysics, Huazhong University of Science and Technology, Wuhan 430074, China

**Keywords:** capacitive sensor, area-changed, parasitic capacitance, fringe effect, sensitivity improvement, micro accelerometer

## Abstract

Encoder-like micro area-changed capacitive transducers are advantageous in terms of their better linearity and larger dynamic range compared to gap-changed capacitive transducers. Such transducers have been widely applied in rectilinear and rotational position sensors, lab-on-a-chip applications and bio-sensors. However, a complete model accounting for both the parasitic capacitance and fringe effect in area-changed capacitive transducers has not yet been developed. This paper presents a complete model for this type of transducer applied to a high-resolution micro accelerometer that was verified by both simulations and experiments. A novel optimization method involving the insertion of photosensitive polyimide was used to reduce the parasitic capacitance, and the capacitor spacing was decreased to overcome the fringe effect. The sensitivity of the optimized transducer was approximately 46 pF/mm, which was nearly 40 times higher than that of our previous transducer. The displacement detection resolution was measured as 50 pm/√Hz at 0.1 Hz using a precise capacitance detection circuit. Then, the transducer was applied to a sandwich in-plane micro accelerometer, and the measured level of the accelerometer was approximately 30 ng/√Hz at 1Hz. The earthquake that occurred in Taiwan was also detected during a continuous gravity measurement.

## 1. Introduction

Displacement transducers are key components of many precise imaging and fabrication machines, with various uses such as detecting the displacement of the probe in an AFM (Atomic Force Microscope), pre-focusing the electron microscopes in testing and examining the wafers, and measurement of the proof mass displacement in an accelerometer. Because the sensor characteristics can define the linearity, sensitivity, and speed of the machine, the sensor performance is a foremost consideration [1,2]. A precise accelerometer is considered a key device in inertial surveying systems, which have been widely used for geology, resource exploration, gravity-aided navigation and earthquake detection [3]. An ultra-high-sensitivity accelerometer is required due to the weak variations of gravity signals. Silicon-based MEMS (micro-electromechanical system) technology has been proven to be a feasible approach toward high-resolution inertial sensors with a stable mechanical structure, low structure dissipation, small size and potentially low cost [4,5,6,7].

The displacement transducer is also a key component of many accelerometers [1]. It translates the displacement variation of the proof mass caused by input acceleration into other signals, such as capacitance [8,9], light intensity and current [10,11]. Capacitive sensors are one of the most commonly used displacement transducers because of their low cost, excellent linearity, high resolution and large bandwidth range [12]. Area-changed transducers have better linearity and a larger dynamic range than gap-changed capacitive transducers. Because the total noise of micromachined accelerometers is typically dominated by electronic noise, increasing the sensitivity of the displacement transducers is an efficient way to improve the resolution of the accelerometer [13]. Reducing the air gap is beneficial for both gap-changed transducers and area-changed transducers; however, the range of gap-changed transducers will be reduced for the open-loop work condition, whereas that of area-changed transducers remains the same. This difference illustrates that area-changed transducers are advantageous in terms of their potential high sensitivity when an appropriate range is needed.

To increase the sensitivity of area-changed transducers, the transducer is divided into a series of parallel-connected encoder-like capacitors, which increases the sensitivity by the number of capacitors. As a result, encoder-like area-changed capacitance has been widely used in various micromachined devices, such as precise displacement sensors [14], rotational position sensors [15], lab-on-a-chip applications (e.g., localization of bio particles) [16] and bioengineering applications (e.g., positioning of micro mirrors for laser surgery and dose control for implantable drug delivery systems) [17]. Due to their many advantages, encoder-like area-changed transducers have also been used in different types of precise inertial sensors, exhibiting low noise and a large dynamic range [18,19,20]. However, because the area of electrodes is increased to achieve a higher sensitivity, parasitic capacitances are regarded as a considerable problem [21,22]. Additionally, when the number of separated transducers increases, the dimension of electrodes in the sensitive direction will be comparable to the capacitor spacing; this will break the condition of the ideal parallel-plate model and strengthen the fringe effect. Earlier literature has focused on the theoretical calculation of the capacitance comprising two plates considering the fringe effect [23], precise control of the air gap to increase the sensitivity [24], the effects of parameters on performance, drift compensation [25] and materials and methods for overcoming parasitic capacitance [26,27]. However, a model of encoder-like area-changed transducers accounting for both the fringe effect and parasitic capacitances has not yet been developed.

When we fabricated and tested the first generation of micro accelerometers using an area-changed capacitive transducer in our laboratory, the calibrated sensitivity was nearly 1/24 th of the sensitivity calculated based on the parallel-plane capacitor model. This low sensitivity led us to further investigate the factors affecting the sensitivity of the transducer. This paper reports on the influences of both fringe capacitances and parasitic capacitance in a low-noise micro accelerometer using encode-like area-changed transducers. Theoretical calculations, simulations and experiments are used to quantitatively evaluate the influence. The results revealed that parasitic capacitance and fringe effect significantly decrease the sensitivity, and accurately reflected the discrepancies between the calibrated and calculated sensitivities of the first-generation accelerometer. The model was subsequently used to guide the optimization methods. With a significant increase in sensitivity, the transducer was applied to the micro accelerometer. The second generation of the micro accelerometer achieved nano-g resolution. Moreover, the model is also valuable for other capacitive transducer applications, such as rotational position sensors, lab-on-a-chip applications and bio-sensors.

## 2. Theoretical Analysis, Simulations and Experiments

**Figure 1:**
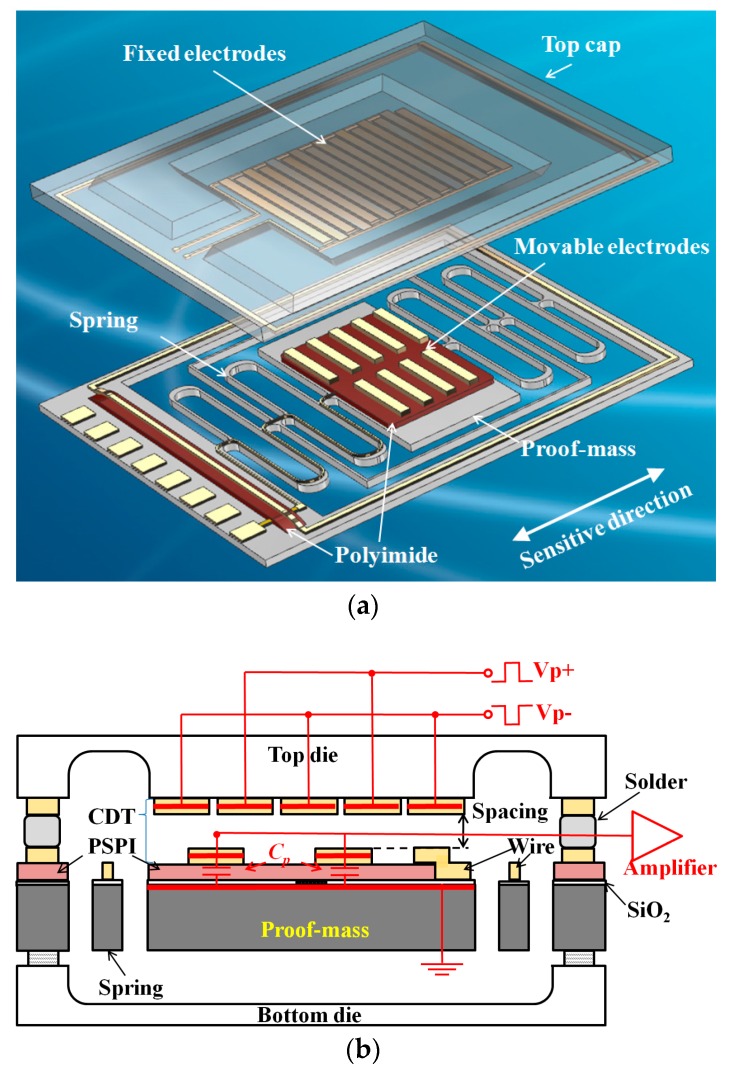
Schematic of the micro accelerometer using an encoder-like area-changed transducer. (**a**) Structure of the capacitive accelerometer. The spring-mass system is also used as a micro position actuator for the calibration of the capacitive transducer; (**b**) Section view of the capacitive accelerometer, the electrodes on the top die and the proof-mass die form the capacitive displacement transducer (CDT); a photosensitive polyimide (PSPI) is inserted for reducing the parasitic capacitance.

A schematic of a micro accelerometer using an encoder-like area-changed capacitive displacement transducer is shown in Figure 1a. The sandwich-type structure of the MEMS consists of three parts: a top die, a silicon middle die with a spring-mass structure and a bottom die for mechanical support and hermetical packaging. The movable electrodes on the proof mass and the fixed electrodes on the top die form the capacitive transducers after packaging through the solder reflow process. First, the input acceleration is sensed by the spring-mass system. The proof mass moves with the displacement relative to the frame and top die. Then, the displacement is translated to capacitance variation by the capacitive transducers; this variation is detected by the capacitance detection circuit. To avoid noise at low frequencies and obtain a differential output, the electrodes on the top die are driven by two AC signals with a phase difference of 180°. This forms an AC bridge together with output electrodes on the proof mass. The electrodes on the proof mass are insulated from the silicon substrate electrically by a SiO_2_ layer with a thickness of 200 nm. The metal layer of electrodes and silicon substrate form a parasitic capacitance that is considerably larger than the detected capacitance (Figure 1b).

### 2.1. Parasitic Capacitance

#### 2.1.1. Model and Calculations

**Figure 2:**
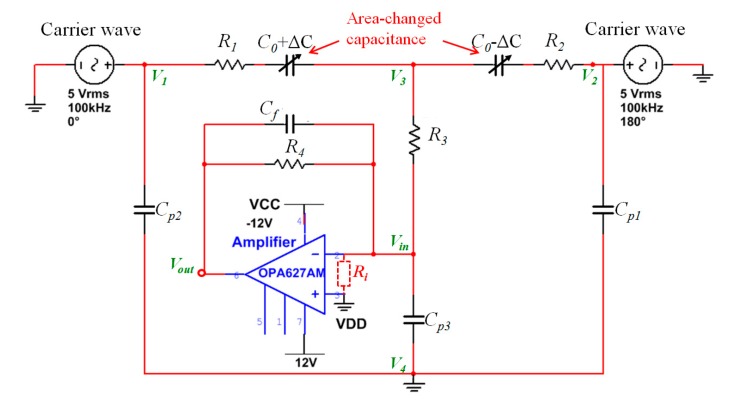
Model of the transducer including parasitic capacitance, where *C*_0_+*ΔC* and *C*_0_-*ΔC* are the differential area-changed capacitances, *C_p_*_1_ and *C_p_*_2_ are the parasitic capacitances of the electrodes on the top die, *C_p_*_3_ is the parasitic capacitance between the electrodes on the proof mass and silicon substrate, and *R*_1_, *R*_2_ and *R*_3_ are the resistances of the deposited wires for signal transmission, *C_f_* and *R*_4_ are the electron devices of the amplifier circuit. The silicon substrate connects to the ground through ohmic contact to overcome influences of *C_p_*_1_ and *C_p_*_2_ on the sensitivity and offset.

The parasitic capacitance was firstly analyzed. The model of the encoder-like area-changed capacitive transducer including parasitic capacitance and the front-end operational amplifier was built based on actual parameters, as shown in Figure 2.

For the ideal model of an amplifier, according to the “virtual short” feature, the input voltage of the amplifier (*V_in_*) equals zero and the input impedance is infinite. Because the silicon substrate is connected to the ground, no current transfers through the parasitic capacitances. The parasitic capacitance has no influence on the output of the amplifier. However, the ideal “virtual short” cannot be achieved in practice, and thus, the input voltage is no longer equal to zero. According to Ohm's law and the feature of amplifiers,

(1) $$\left\{ \begin{array}{l} \frac{1}{C_{f}s+\frac{1}{R_{4}}}=\frac{V_{out}-V_{in}}{I_{in}}\\ V_{out}-AV_{in}=0\\ R_{i}=\frac{V_{in}}{I_{in}} \end{array}\right.$$

where *s* is the complex number of the Laplace transform, *A* is the gain of the amplifier and *R_i_* is the input impedance of the amplifier. *R_i_* can be expressed as:

(2) $$R_{i}=\frac{1}{\left(A-1)(C_{f}s+\frac{1}{R_{4}}\right)}$$

When the parasitic capacitance (*C_p_*_3_) connects to the impedance of the amplifier in parallel, it will decrease the effective impedance (*R_i_*’) as follows:

(3) $$R_{i}{}^{\prime }=\frac{1}{\frac{1}{R_{i}}+C_{p}{}_{3}s}$$

As a result, the component voltage of the effective impedance (*V_in_*) is reduced for the same variation of input capacitance, resulting in a decreasing output (*V_out_*), which is defined by A*V_in_. The attenuation coefficient (*K_p_*), which is defined by the ratio of the output with parasitic capacitance and the output without capacitance, should be

(4) $$K_{p}=\frac{V_{out}{}^{\prime }}{V_{out}}=\frac{R_{i}{}^{\prime }}{R_{i}}$$

where *V_out_’* is the output of amplifier with parasitic capacitance and *V_out_* is the output of the amplifier without parasitic capacitance. In practice,

(5) $$\left\{ \begin{array}{l} C_{f}s>>\frac{1}{R_{4}}\\ A>>1 \end{array}\right.$$

From Equation (2) to Equation (5), the influence on sensitivity can be simplified as:

(6) $$K_{p}=\frac{1}{1+\frac{C_{p}{}_{3}}{AC_{f}}}$$

A larger parasitic capacitance will lead to a greater decrease in sensitivity. An extreme situation is as follows:

(7) $$C_{p}{}_{3}>>AC_{f}K_{p}=\frac{AC_{f}}{C_{p}{}_{3}}$$

Here, the sensitivity decrement is inversely proportional to the capacitance. An alternative extreme situation is:

(8) $$C_{p}{}_{3}<<AC_{f}\;K_{p}=1$$

In this case, parasitic capacitance has no influence on the output when it is considerably lower than the product of the amplifier gain and feed-back capacitance. It should be noted that the parasitic capacitance is large for capacitive transducers in a high-precision micro accelerometer. As a result, Equation (8) is not applicable, and the influence of the parasitic capacitance is remarkable.

#### 2.1.2. Simulation and Experiments

Both simulations and experiments were conducted to verify the model and the calculations presented above. The simulation was performed with Multisim schematic capture simulation tools based on the model shown in Figure 2. All of the parameters of the electrics were assigned according to the measurements using an LCR meter except for the amplifier, whose parameters were based on the chip datasheet of OPA627AM. Table 1 lists the parameters of the electrics.

Several fixed capacitors were employed to quantify the effect of parasitic capacitances on the sensitivity experimentally. Capacitors with capacitances of 2.5 and 5.5 pF were connected to the input terminal of the amplifier acting as the differential area-changed capacitances. Capacitors with capacitances of 100, 560 and 1000 pF were connected in parallel from the input terminal to the ground successively to act as the parasitic capacitances.

To decrease parasitic capacitances, 3-μm-thick PSPI, which is extremely stable after an annealing process, was inserted between the metal layer and silicon substrate to increase the thickness of the dielectric layer. The compatibility of the PSPI during the deep reactive ion etching (DRIE) process was also solved [28,29].

### 2.2. Capacitance of the Fringe Effect

**Figure 3:**
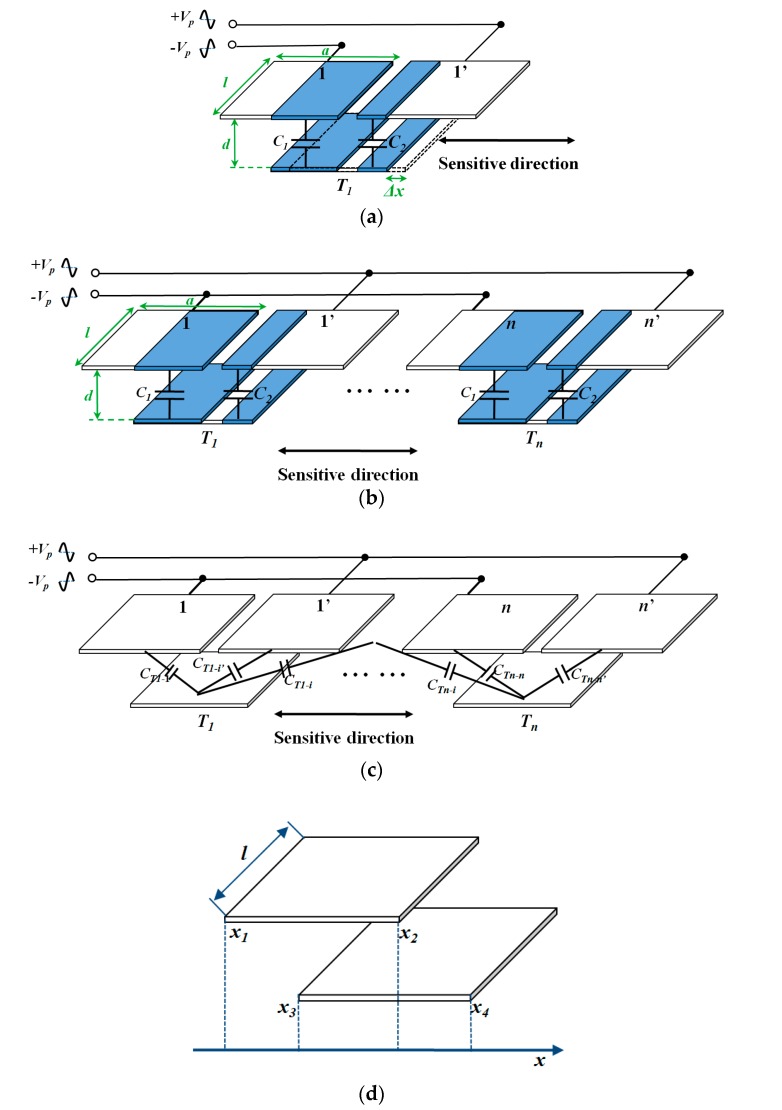
Model of the area-changed capacitive transducer. (**a**) Single area-changed capacitive displacement transducer; (**b**) Encoder-like area-changed capacitive transducer using the ideal plane-parallel capacitor model; (**c**) Encoder-like area-changed capacitive transducer model considering the fringe effect; (**d**) Model of Heerens’s formula for calculation fringe effect.

Fringe effect was then analyzed. The model of the area-changed capacitive transducer is shown in Figure 3.

Figure 3a shows a single differential area-changed capacitive displacement transducer. The electrode 1, electrode 1’ are placed on the fixed top die, while the electrode *T*_1_ is placed on the movable proof mass. The transducer can be treated as an ideal plane-parallel capacitor when the dimensions of the electrodes (*a*, *l*) are considerably larger than the plate spacing (*d*) of the capacitor. As a result, only the capacitance of the facing area is considered. When the electrode on proof mass has a displacement Δ*x* along the sensitive direction, the facing area to both electrode 1 and electrode 1’ changes with opposite value,

(9) $$\Delta C_{2}=-\Delta C_{1}=\frac{\varepsilon_{0}\varepsilon_{r}l\Delta x}{d}$$

where *l* is the length of the electrode, *d* is the spacing between electrode plates, and Δ*x* is the displacement to be detected. The output of the differential transducer is:

(10) $$\Delta C_{s}=\Delta C_{1}-\Delta C_{2}=\frac{2\varepsilon_{0}\varepsilon_{r}l\Delta x}{d}$$

The differential output rejects common mode noise while doubling the sensitivity.

As shown in Figure 3b, the encoder-like area-changed capacitive transducer is formed by series parallel connected single differential capacitive transducers. The capacitance variation is the sum of all the single transducers.

(11) $$\Delta C=n\Delta C_{s}\approx 2n\frac{\varepsilon_{0}\varepsilon_{r}l}{d}\Delta x$$

where *n* is the number of arrayed differential capacitors.

According to Equation (11), *n* should be as large as possible to increase the sensitivity. However, a larger *n* requires a smaller dimension of electrodes in the sensitive direction (*a*), which will violate the dimension conditions of the ideal plane-parallel capacitor model. Therefore, the fringe effect must be considered. As shown in Figure 3c, the capacitances between any two electrodes should be included, not simply the capacitance of the facing areas.

Heerens derived the formula for the calculation of a biplanar capacitor considering fringe effect [23]. As shown in Figure 3d, the capacitance between the two electrodes is:

(12) $$C=\frac{\varepsilon_{0}\varepsilon_{r}l}{\pi }\ln\left\{\frac{\cosh[\frac{\pi }{2d}\left(x_{2}-x_{3}\right)]\cosh[\frac{\pi }{2d}\left(x_{1}-x_{4}\right)]}{\cosh[\frac{\pi }{2d}\left(x_{1}-x_{3}\right)]\cosh[\frac{\pi }{2d}\left(x_{2}-x_{4}\right)]}\right\}$$

Setting *x*_3_ as the zero point in the x axis and put in the parameters of electrode, the formula can be simplified as:

(13) $$C=\frac{\varepsilon_{0}\varepsilon_{r}l}{\pi }\ln\left\{\frac{\cosh[\frac{\pi }{2d}\left(x+a\right)]\cosh[\frac{\pi }{2d}\left(x-a\right)]}{\cosh^{2}\left(\frac{\pi x}{2d}\right)}\right\}$$

where *x* = *x*_1_ is the relative position between the two electrodes in the sensitive direction. For electrode *T*_1_ on the proof mass, the capacitance of *T*_1_ to electrodes on top die:

(14) $$C_{T_{1}}=\left(C_{T_{1}-1}-C_{T_{1}-1^{\prime }}\right)+\left(C_{T_{1}-2}-C_{T_{1}-2^{\prime }}\right)+\ldots \left(C_{T_{1}-i}-C_{T_{1}-i^{\prime }}\right)\ldots +\left(C_{T_{1}-n}-C_{T_{1}-n^{\prime }}\right)$$

where $C_{T_{1}-1}$,$C_{T_{1}-1^{\prime }}$……$C_{T_{1}-n}$,$C_{T_{1}-n^{\prime }}$ can be calculated using Equation (13). 

**Figure 4:**
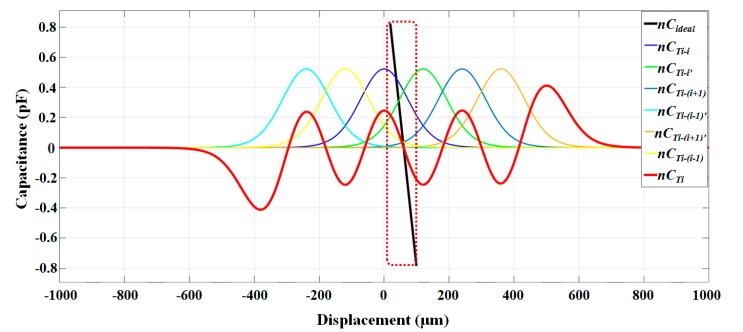
Calculation of the capacitance between electrode *T_i_* on the proof mass and 6 nearest electrodes on the top die considering the fringe effect. The capacitor spacing was set as 120 μm. $C_{ideal}$ (black line) is the theoretical output using an ideal plane-parallel capacitor model. $C_{T_{i}}$ (red line) is the theoretical output considering the fringe effect, which is the total contribution of $C_{T_{i}-i}$, $C_{Ti-i^{\prime }}$, $C_{T_{i}-i+1}$, $C_{Ti-{\left(i+1\right)}^{\prime }}$, $C_{T_{i}-i-1}$, $C_{Ti-{\left(i+1\right)}^{\prime }}$. The number of capacitors is multiplied.

We analyzed the fringe effect of the electrode *T_i_* using our model as an example. Assume that the original position of electrode *T_i_* is facing the electrode *i* on the top die completely. When the displacement varies from 20 to 100 μm, the variations of $C_{T_{i}-i}$, $C_{Ti-i^{\prime }}$, $C_{T_{i}-i+1}$,$C_{Ti-{\left(i+1\right)}^{\prime }}$, $C_{T_{i}-i-1}$,$C_{Ti-{\left(i-1\right)}^{\prime }}$ were calculated using Equation (13), as shown in Figure 4.

It can be seen from Figure 4 that the slop of $C_{ideal}$ is larger than $C_{T_{i}}$, which means that when considering fringe effect, the theoretical sensitivity will be less than using a plane-parallel capacitor model.

For all the electrodes on the proof mass, considering the period distribution of the electrodes, the total capacitance is:

(15) $$C_{e}=C_{T_{1}}+C_{T_{2}}+\ldots \ldots +C_{T_{n}}=nC_{T_{1}}$$

The capacitance variation caused by displacement should be:

(16) $$\Delta C_{e}=\frac{dC_{e}}{dx}\Delta x$$

As a result, the actual output differs from that of the plane-parallel capacitor. The influence of the fringe effect is defined by:

(17) $$K_{e}=\frac{\Delta C_{e}}{\Delta C}$$

A finite element analysis (FEA) was conducted using Ansoft Maxwell 3D field simulator to verify the calculation. The model was built based on the actual dimensions and material properties. The simulating region was set to be three times larger than the model such that both the capacitance of the faced areas between electrodes on the top die and proof mass and the fringe effect capacitances caused by any non-faced areas of electrodes were included.

Considering the influence on sensitivity from both parasitic capacitance and the fringe effect, the sensitivity of the model of the encoder-like area-changed capacitive transducer would be:

(18) $$\frac{\Delta C}{\Delta x}=2n\frac{\varepsilon_{0}\varepsilon_{r}l}{d}\cdot K_{p}\cdot K_{e}$$

This equation was used to calculate the sensitivity of the complete encoder-like area-changed capacitive transducer model. It should be mentioned that electrodes on the top die are designed to be a litter more to avoid nonlinearity from the edges of the transducers.

### 2.3. Fabrication and Testing

**Figure 5:**
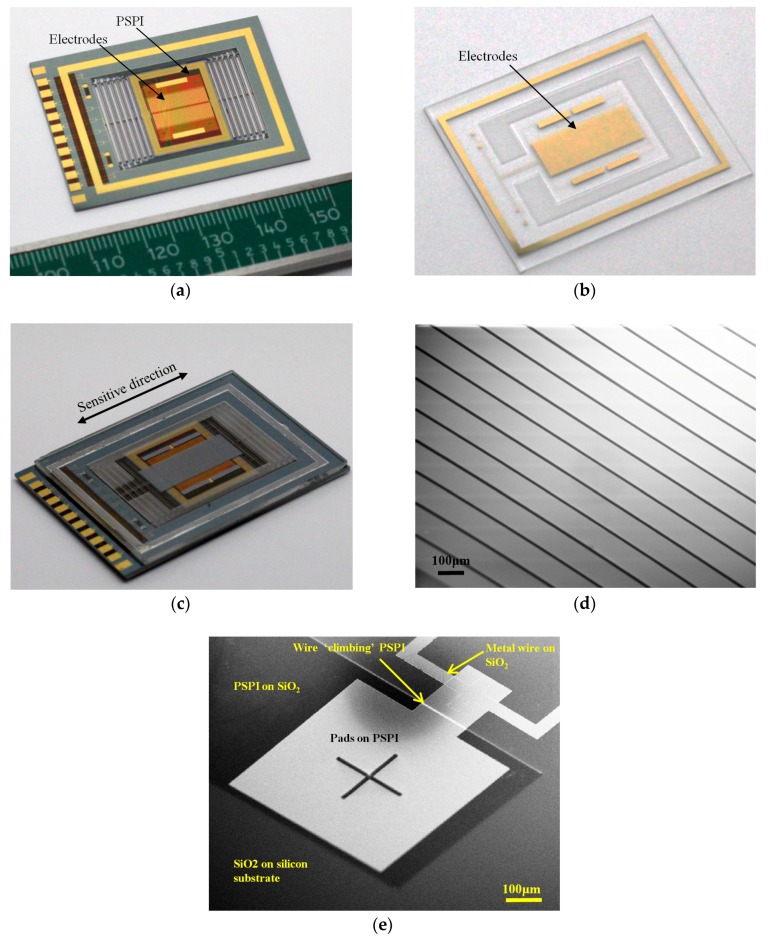
Fabricated micro accelerometer. (**a**) Electrodes on the spring-mass structure; (**b**) Electrodes on the top die; (**c**) Packaged micro accelerometer in which the electrodes on the top die and spring-mass structure form the area-changed capacitive transducer; (**d**) SEM photograph of the electrodes; (**e**) Additional PSPI between the metal layer and silicon substrate, the wire climbs the edge of the PSPI for transferring the signal from the electrodes and pads on PSPI.

The micro capacitive transducer and accelerometer were fabricated in our clean room [30], as shown in Figure 5.

The electrodes on both the top die and the spring-mass die were deposited by electron beam evaporation and patterned by a lift-off process. Then, through-wafer etching was performed using an ICP system to obtain the bulk spring-mass system. The spring-mass system of the accelerometer was used as a precise micro position actuator to test the sensitivity of the capacitive transducer. Combining the spring-mass system and the capacitive transducer also makes an accelerometer. For the optimized transducer, a PSPI layer with a thickness of 3 μm was spin coated before the electrodes were fabricated. The gentle slope of the PSPI layer is beneficial to the connection of metal layers on PSPI and SiO_2_. Finally, the top die and spring-mass die were bonded by a solder reflow process. The electrodes on both die formed the capacitive transducer. In order to control the thickness of the reflow which is critical for the gap of capacitors, precise feelers were inserted in the gap to make stoppers. The key parameters of the fabricated micro capacitive transducer and accelerometer are summarized in Table 2.

**Figure 6:**
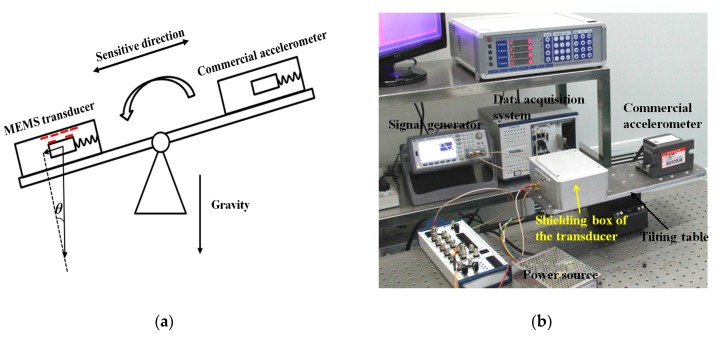
Calibration system. (**a**) Principle of the sensitivity calibration system using a tilting table; (**b**) Setup of the system. The capacitive transducer was set inside the shielding box.

The sensitivity calibration system of the capacitive transducer is shown in Figure 6. The capacitance of the transducer was measured by a capacitive detecting printed circuit board (PCB) inside a shielding box. The wave carriers to both driving electrodes arrays, which have a zero-to-peak amplitude of 5V and a frequency of 100 kHz, were produced by a signal generator. The silicon substrate was connected to the ground of the PCB through an ohmic contact pad with a resistance of less than 1Ω [31]. After demodulation and low-pass filtering, the output data was acquired with an NI 6281 card. To calibrate the sensitivity, a tilting table was used to set a micro parallel displacement between electrodes on the proof mass and top die. A commercial accelerometer (CMG-5U, Guralp) measured the input acceleration, which enables the displacement to be quantified as:

(19) $$\Delta x=\frac{\Delta a}{\omega^{2}}$$

where Δ*a* is the output of the commercial accelerometer and *ω* is the angular frequency of the spring-mass system, which can be defined as 2π*f*. The consecutive tilting angle was set to be approximately ±1.5°, which will supply a movement of approximately ±30 μm for the proof mass. The outputs of the capacitive circuit and commercial accelerometer were acquired with a sampling rate of 100 Hz.

## 3. Results

**Figure 7:**
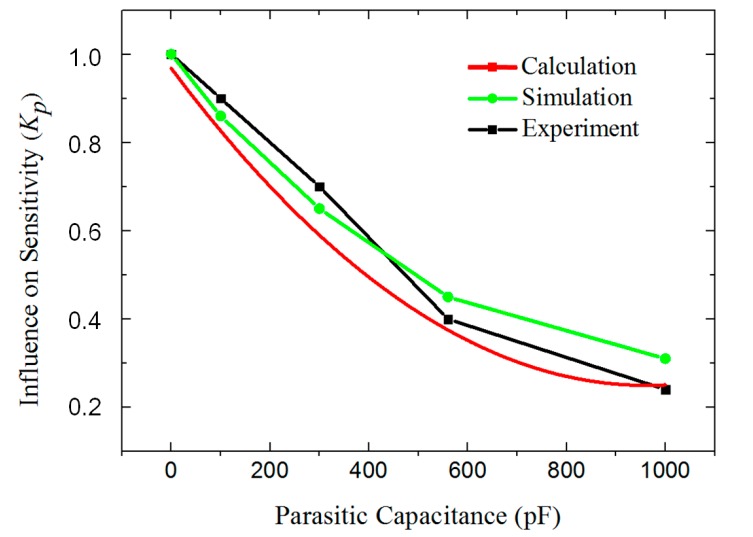
Influence of the parasitic capacitance on the sensitivity, which is defined by the ratio of the output with parasitic capacitance and the output without parasitic capacitance under the same input.

The theoretical, simulated and experimental results of the decrease in sensitivity caused by the parasitic capacitance are shown in Figure 7. The sensitivity of the transducer is negatively correlated with the parasitic capacitance. The simulation and experiment corresponded well with the calculations based on Equation (6).

The parasitic capacitance of the original transducer is approximately 1000 pF. After being optimized with an additional PSPI layer, the parasitic capacitance was limited to approximately 406 pF. As a result, the sensitivity is increased nearly two times according to Figure 7.

**Figure 8:**
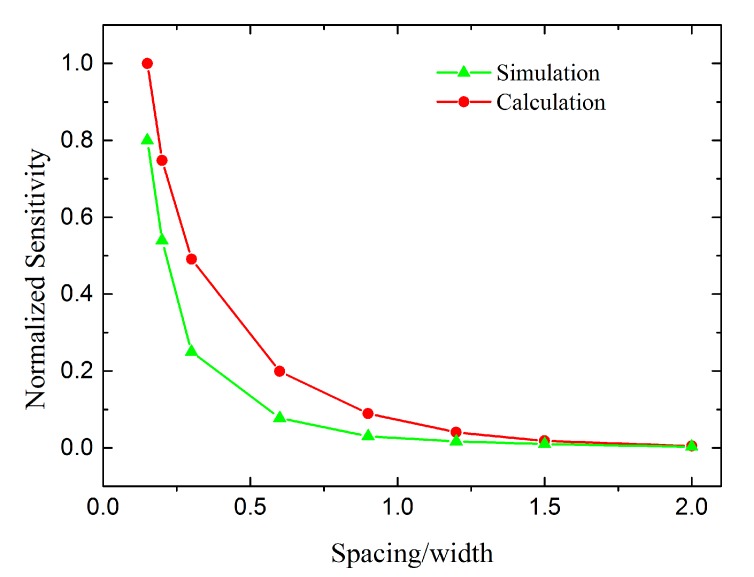
Calculation and Finite Element Modeling (FEM) results for the sensitivity of the transducer considering the fringe effect with different ratios of the capacitor spacing and electrode width (*d*/*a*).

Figure 8 shows the calculation and Finite Element Modeling (FEM) results for the sensitivity of the transducer considering the fringe effect. The width is constant while the capacitor spacing varies from 15 to 200 μm. The sensitivity of the transducer with a spacing of 15 μm was set as the normalizing reference. The simulated and calculated results show the same tendency. 

**Figure 9:**
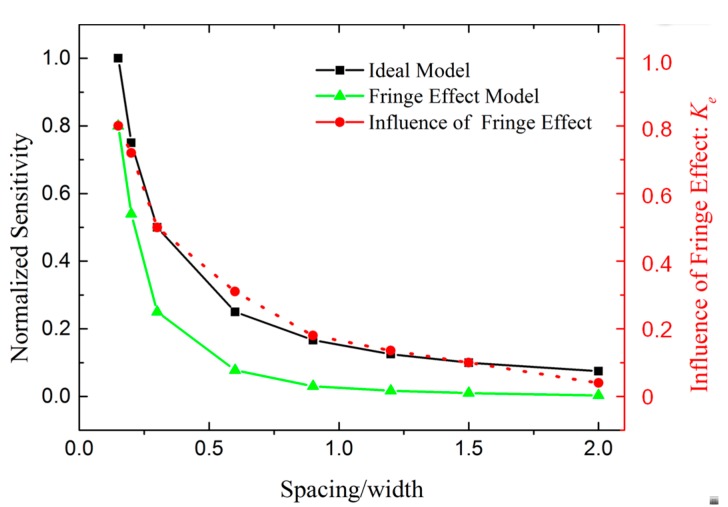
FEM results showing the decreased insensitivity caused by the fringe effect. The black line is the normalized sensitivity of the plane-parallel capacitor from an ideal model. The green line is the normalized sensitivity of the area-changed capacitance considering the fringe effect. The red line is the influence of the fringe effect (*K_e_*), defined by the value of the points on the green line divided by the points on the black line.

The decreased insensitivity caused by the fringe effect is shown in Figure 9. The curves reveal that the fringe capacitance induces a decrease in the sensitivity. The decrement becomes particularly significant when the capacitor spacing becomes larger than the width of a single electrode. 

The capacitor spacing of the capacitive transducer in the micro accelerometer was then optimized from 90 μm to 20 μm; according to Figure 9, this optimization should increase the sensitivity by 18 times based on both the reduction in the fringe effect and the decreased plate spacing. The fringe effect still reduces the sensitivity by 30% after the optimization. Further optimization of the spacing is limited by the warpage on the surface of the spring-mass system.

For our first-generation micro accelerometer, the parasitic capacitance was approximately 1000 pF, the capacitor spacing was 90 μm, and the calibrated sensitivity was only 1.1 pF/mm. The theoretical sensitivity of the parallel-plate capacitor model (Equation (11)) is 26 pF/mm, which did not correspond with the experimental results. However, using our complete model (Equation (18)), *K_e_* is approximately 0.18 and *K_p_* is approximately 0.25, according to Figure 7 and Figure 9. The sensitivity is 1.2 pF/mm, which corresponds better with the experimental results and thus verifies our theory.

**Figure 10:**
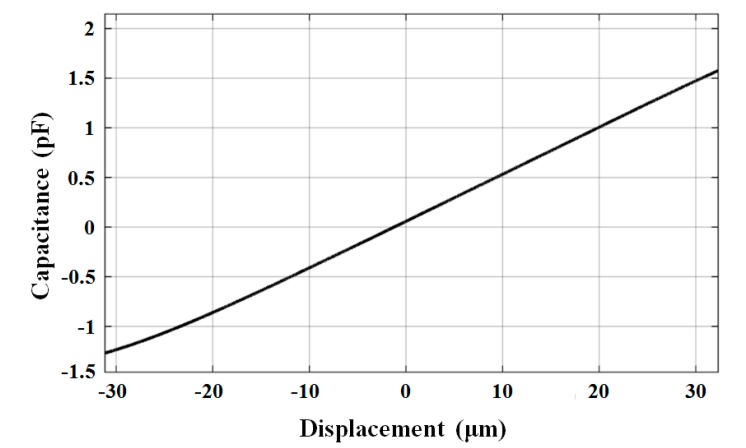
Sensitivity calibration of the capacitive transducer.

The calibration curve of the optimized capacitive transducer is shown in Figure 10. The sensitivity was measured as 46 pF/mm. Using the capacitive readout circuit with a noise floor of 2 × 10^−6^ pF/√Hz at 0.1 Hz, a displacement measurement resolution of 50 pm/√Hz was achieved at 0.1 Hz. The displacement resolution can be further optimized by using a better readout circuit. The linearity error of the transducer within ±10 μm was 0.04% of the measurable range. The offset was from the original displacement caused by installation error and packaging error.

**Figure 11:**
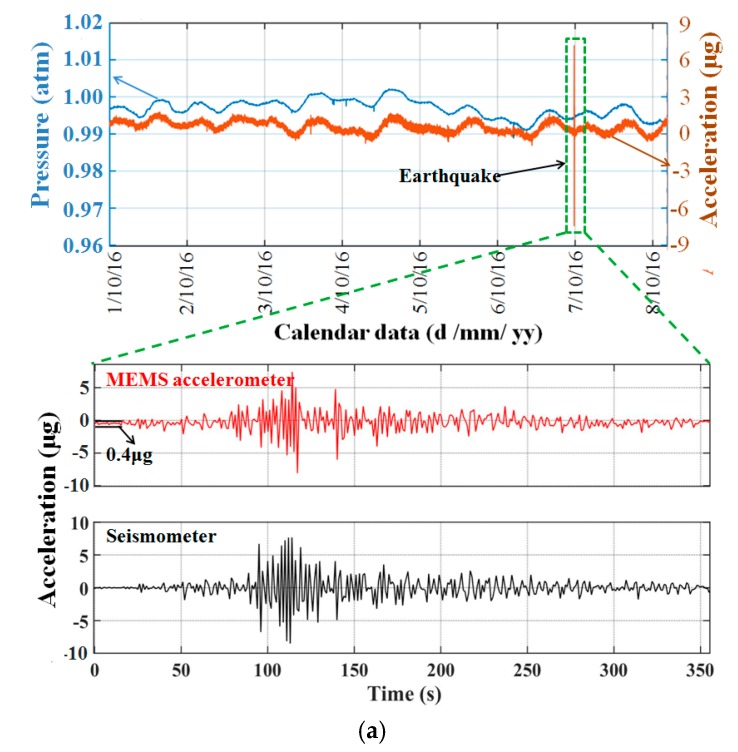

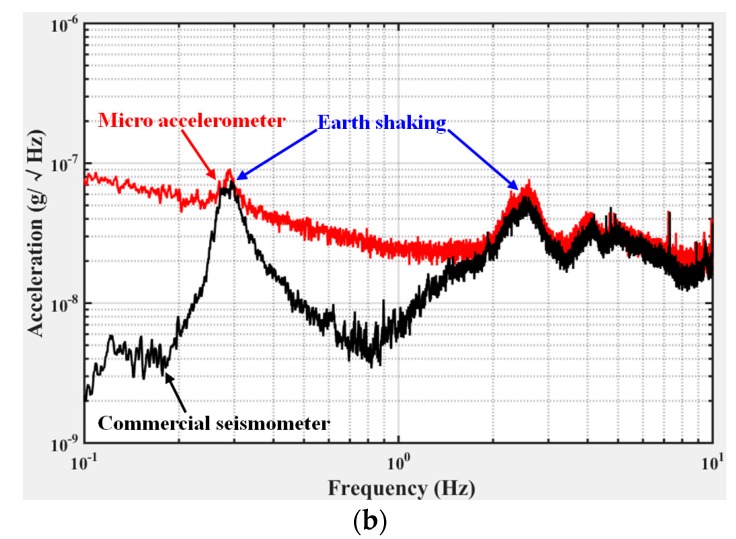
Performance of the micro accelerometer using the optimized area-changed transducer. (**a**) Static output of the area-changed capacitive micro accelerometer for eight days. A Ms5.9 earthquake that occurred in Taiwan at 23:51 06/10/2016 (UTC-8) was detected. The details of the earthquake detected by our micro accelerometer and the commercial seismometer show good agreement. (**b**) Noise-limited resolution of the optimized accelerometer.

The optimized capacitive transducer was applied to the micro accelerometer. A static measurement was performed in our cave laboratory under a quiet environment for earthquake monitoring from 1 October 2016 to 8 October 2016 (UTC-8). A commercial seismometer (CMG-3EPS, Guralp) was installed adjacent to the micro accelerometer as a reference. The output data of the accelerometer is shown in Figure 11a. The unusual vibration was proven to be the Ms5.9 earthquake that occurred in Taiwan at 23:51 6 October 2016 (UTC-8). The vibration was also detected by the commercial seismometer with a similar amplifier and profile. The continuous output of the accelerometer shows a strong correlation with the variation of air pressure. This correlation with air pressure is probably because when the air pressure of the outside environment changed, a deformation of the top die occurred as the pressure inside the sensors remained the same as the hermetic sealing, then the spacing of the capacitive transducer changed causing the change in the capacitance. It also implies the possibility of improving the long-term stability by alleviating the pressure interference. The experimentally measured noise-limited resolution of the optimized accelerometer is shown in Figure 11b. The resolution was approximately 30 ng /√Hz at 1 Hz. The peak caused by the earth shaking was also detected by the commercial high-resolution seismometer (Guralp, 3ESPC), which indicates that the noise floor of the MEMS was better than the background noise in our laboratory at frequencies of approximately 0.3 Hz and 2.5 Hz.

## 4. Conclusions

This paper presents a novel, complete model of an encoder-like area-changed capacitive transducer considering both parasitic capacitance and the fringe effect. The model was quantitatively verified by simulations and experiments. Optimization methods were also proposed and performed. The sensitivity of the optimized micro capacitive transducer is 46 pF/mm, and the displacement resolution is 50 pm/√Hz at 0.1 Hz. This transducer is one of the most sensitive capacitive displacement transducers reported to date. The study is instructive for the design of encoder-like area-changed capacitive transducers for application to precise displacement or position measurements with a large dynamic range. The transducer was applied to a micro accelerometer and achieved less than 30 ng /√Hz of noise at 1 Hz. The accelerometer measured the Ms5.9 earthquake that occurred in Taiwan, which is approximately 1000 km away from the laboratory, with a high signal noise Ratio (SNR). The study can also serve as a reference for other applications, such as lab-on-a-chip applications, including the localization of bio particles, and bioengineering applications, including the positioning of micro mirrors for laser surgery and dose control for implantable drug delivery systems.

## Figures and Tables

**Table 1:** Parameters of the electrics.

Component	Value	Unit
Resistance of the wire (*R*_1_, *R*_2_)	20	Ohm
Original capacitance (*C*_0_)	4	pF
Resistance of the wire (*R*_3_)	770	Ohm
Parasitic capacitance (*C_p_*_1_, *C_p_*_2_)	100	pF
Parasitic capacitance pre-optimization (*C_p_*_3_)	1000	pF
Parasitic capacitance post-optimization (*C_p_*_3_*’*)	406	pF
Feedback capacitance (*C_f_*)	2	pF
Feedback resistance (*R*_4_)	100 M	Ohm
Gain of the amplifier at 100 kHz (*A*)	158	V/V

**Table 2:** Summary of the key parameters of the accelerometer.

Component	Value	Unit
Thickness of SiO_2_	0.2	μm
Thickness of electrodes	0.4	μm
Thickness of the PSPI	3	μm
Solder bonding thickness pre-optimization	90	μm
Solder bonding thickness post-optimization	20	μm
Dimension of a single electrode	0.1 × 0.3	mm
Width of spring	42	μm
Length of spring	9	mm
Number of springs on both side of proof mass	12	pair
Number of arrayed capacitors (*n*)	45	-
Length of the electrode (*l*)	3	mm
Width of a single electrode (*a*)	0.1	mm
Capacitors spacing pre-optimization (*d*)	90	μm
Capacitor spacing post-optimization (*d’*)	30	μm
Thickness of the silicon substrate	500	μm
Mass of the proof mass (*m*)	0.4	g
Frequency of the spring-mass structure (*f*)	15	Hz
